# Performing photosynthesis without β-carotene

**DOI:** 10.7554/eLife.63584

**Published:** 2020-11-03

**Authors:** Alison Telfer

**Affiliations:** Department of Life Sciences, Imperial CollegeLondonUnited Kingdom

**Keywords:** caroteonoid, photosynthesis, photosystems, light harvesting, *N. tabacum*, Other

## Abstract

Research on mutant tobacco plants shows that a pigment called β-carotene is not necessary for photosynthesis.

**Related research article** Xu P, Chukhutsina VU, Nawrocki WJ, Schansker G, Bielczynski LW, Lu Y, Karcher D, Bock R, Croce R. 2020. β-carotene is not essential for photosynthesis in higher plants. *eLife* **9**:e58984. doi: 10.7554/eLife.58984

Carotenoids are organic pigments that have an important role in photosynthesis, but they remain hidden for most of the year, only revealing their wonderful yellow and orange colours during the autumn, when chlorophyll – the pigment that makes plants green – is degraded. The most common carotenoids involved in photosynthesis are carotenes (which contain 40 carbon atoms and a variable number of hydrogen atoms) and xanthophylls (which are oxidised carotenes).

The carotenoids involved in photosynthesis have two major roles – to harvest light and to prevent damage when the plant is overexposed to sunlight (Frank and Cogdell, 1996). The first role involves absorbing visible light and transferring the energy to chlorophyll. The second involves inactivating or 'quenching' an excited state of chlorophyll called a triplet state: if this state is not quenched it reacts with oxygen molecules to form singlet oxygen – an excited state of oxygen that can destroy chlorophyll and cause damage to proteins and lipid membranes in the photosynthetic machinery of the plant (Krieger-Liszkay, 2005).

Photosynthesis typically happens in pigment protein complexes called photosystems. At the heart of the photosystem is the reaction centre, an enzyme that uses light to reduce molecules. This reaction centre is surrounded by light-harvesting complexes, protein assemblies that enhance the absorption of light. These assemblies contain two types of carotenoids: β-carotene, which binds to the reaction centres, and several kinds of xanthophyll, which bind to the light harvesting complexes (Siefermann-Harms, 1985; Telfer, 2002).

Previous research has shown that mutant plants that lack xanthophyll but not β-carotene can still conduct photosynthesis (Tóth et al., 2015). And since β-carotene can be found in every photosynthetic organism, it has long been assumed that this pigment must be essential for photosynthesis. Now, in eLife, Roberta Croce and colleagues at the VU University Amsterdam and the Max Planck Institute of Molecular Plant Physiology – including Pengqui Xu as first author – report that this may not be the case after all (Xu et al., 2020).

The researchers engineered a mutant tobacco plants that lacked β-carotene and contained a xanthophyll called astaxanthin (a pigment that is found in salmon, shrimps and some algae but not plants). Xu et al. discovered that these plants were still able to perform photosynthesis and concluded that β-carotene is not essential for this process. Instead, astaxanthin acted as a substitute for β-carotene and was able to bind to both the reaction centre and the light harvesting complex. Moreover, the photosystems were stable, and the plants were still able to conduct photosynthesis, even though some of the β-carotene binding sites remained empty. Mutant plants were more sensitive to light, which is likely due to the reduced level of β-carotene (and hence a reduced ability to quench the triplet state of chlorophyll).

However, previous research has shown that xanthophylls can also quench chlorophyll in a triplet state if they are close enough. This reaction also leaves the xanthophyll in a triplet state (which decays harmlessly producing heat) and quenching by xanthophylls is faster than the reaction producing singlet oxygen. Xanthophylls located in the light harvesting complexes help to prevent bleaching of chlorophyll at high light intensities (Mozzo et al., 2008; Edge et al., 1997), which is not the case for β-carotene in the reaction centres (Telfer, 2002).

The discovery that a xanthophyll (astaxanthin) can take over from β-carotene in the reaction centres of both photosystems in tobacco mutants raises the question of why, in wild type plants, the reaction centres only bind β-carotene and not a xanthophyll. Is β-carotene simply better at quenching singlet oxygen (Telfer et al., 1994)? This idea seems to be supported by the fact that the rate of irreversible bleaching of chlorophyll was greater the less β-carotene was present in reaction centres, due to a higher number of singlet oxygen molecules being formed there.

The mutant plants of Xu et al. did not grow as fast as wild type plants, nor do they contain as much chlorophyll per unit area of the leaves. Further work is needed to assess the viability and competitiveness of the mutant plant compared to the wild type, which may reveal why native plants use β-carotene and not xanthophylls in their reaction centres. Nevertheless, Xu et al. show that the photosynthetic system can adapt to changes, even if they are unnatural. This could play an important role for redesigning photosynthetic complexes to, for example, improve the productivity of crops.
